# Improving the management of menopause in women with serious mental illness

**DOI:** 10.1192/bjo.2021.500

**Published:** 2021-06-18

**Authors:** Maria-Elena Di Lorenzo, Thomas Reilly, Tom Walker-Tilley, Shubhra Mace

**Affiliations:** 1 South London and Maudsley NHS Foundation Trust; 2 South London and Maudsley NHS Foundation Trust, Institute of Psychiatry, Psychology & Neuroscience, Kings College London

## Abstract

**Aims:**

To improve the diagnosis and management of menopause in women with a serious mental illness in psychiatric services. This will be achieved by developing a questionnaire to systematically assess symptoms related to the menopause, based on NICE guidelines. Women will be offered information and advice, according to these guidelines. Barriers to the assessment or management of the menopause will be identified by piloting the questionnaire on an inpatient female ward.

**Method:**

Women aged 40 years and over, admitted to an acute female in-patient ward in South London and Maudsley NHS Foundation Trust, were interviewed using a structured questionnaire.

**Result:**

In total, 23 eligible women were approached of whom 17 (74%) agreed to take part with mean age 53 years (range 40–67 years). Nine women reported that they had undergone the menopause and four women reported experiencing perimenopausal symptoms. Fifteen women had not previously received information about the menopause. Of the 13 women who had undergone the menopause or were experiencing irregular periods, 7 reported experiencing hot flushes, night sweats and a general change in physical and mental health and four reported a change in mood. Seven women reported that the changes noted may have been related to the menopause over the previous 12 months. Eight women requested further information either in written format or in the form of an information group about the menopause.

**Conclusion:**

We identified women who were admitted to a psychiatric ward who had experienced symptoms related to the menopause that had impacted on their mental and physical health. It was evident that the majority of these women with severe mental illness had not had the opportunity to discuss their symptoms with a healthcare professional in the past and a significant proportion welcomed further information to help make sense of their symptoms. We intend to implement the questionnaire trust-wide with the eventual aim of developing a local guideline to inform the assessment and management of the menopause within our services.

